# Highly Stretchable Double Network Ionogels for Monitoring Physiological Signals and Detecting Sign Language

**DOI:** 10.3390/bios14050227

**Published:** 2024-05-03

**Authors:** Ya Jiang, Shujing Zhao, Fengyuan Wang, Xiaoyuan Zhang, Zhiqiang Su

**Affiliations:** State Key Laboratory of Chemical Resource Engineering, Beijing Key Laboratory of Advanced Functional Polymer Composites, Beijing University of Chemical Technology, Beijing 100029, China; 2021200427@buct.edu.cn (Y.J.); 2021400142@buct.edu.cn (S.Z.); 15832848097@163.com (F.W.)

**Keywords:** double network, ionic liquid, ionogels, strain sensors, sign language detection

## Abstract

At the heart of the non-implantable electronic revolution lies ionogels, which are remarkably conductive, thermally stable, and even antimicrobial materials. Yet, their potential has been hindered by poor mechanical properties. Herein, a double network (DN) ionogel crafted from 1-Ethyl-3-methylimidazolium chloride ([Emim]Cl), acrylamide (AM), and polyvinyl alcohol (PVA) was constructed. Tensile strength, fracture elongation, and conductivity can be adjusted across a wide range, enabling researchers to fabricate the material to meet specific needs. With adjustable mechanical properties, such as tensile strength (0.06–5.30 MPa) and fracture elongation (363–1373%), this ionogel possesses both robustness and flexibility. This ionogel exhibits a bi-modal response to temperature and strain, making it an ideal candidate for strain sensor applications. It also functions as a flexible strain sensor that can detect physiological signals in real time, opening doors to personalized health monitoring and disease management. Moreover, these gels’ ability to decode the intricate movements of sign language paves the way for improved communication accessibility for the deaf and hard-of-hearing community. This DN ionogel lays the foundation for a future in which e-skins and wearable sensors will seamlessly integrate into our lives, revolutionizing healthcare, human–machine interaction, and beyond.

## 1. Introduction

The skin is the largest organ in the human body, serving as both a protective barrier and a sensory organ. It helps to protect the body from external damage, such as UV radiation, pathogens, and mechanical injury. It also senses external stimuli, such as temperature, pressure, and humidity [1,2]. The skin transduces external stimuli like temperature, pressure, and humidity into electrical signals that course through the nerves to the brain, dictating our body’s response. As electronics evolve, e-skins are on the rise, mimicking skin’s intricate functions and promising a future where technology will seamlessly blend with our biology [3,4,5]. E-skins revolutionize human–machine interaction by directly sensing external stimuli (pressure, temperature, humidity) and encoding them into distinct electrical signals (capacitance, resistance, current, voltage) with remarkable accuracy and detail [6].

Bridging the gap between body and technology in real time, sensing materials in wearables translate the world’s whispers (pressure, heat, even moisture) into instant electrical conversations, driving device reactions. Traditional wearables usually shackle themselves to rigid materials like metals and ceramics. These rigid material-based devices lack mechanical flexibility and portability, do not attach well to the human body, and suffer from problems such as inaccurate sensing data and poor sensing accuracy [7]. Researchers have not been constrained by rigid materials, but have developed a series of flexible substrates on this basis, such as polydimethylsiloxane (PDMS)[8,9], polyvinylidene difluoride (PVDF) [10,11], polyethylene (PE) [12], polyimide (PI) [13], and polyethylene terephthalate (PET) [14], giving birth to a generation of soft wearable electronic products. While these substrates are more flexible, many of them are deficient in electrical conductivity. To improve the conductivity of these substrates, conductive nanomaterials need to be added, including carbon nanotubes (CNTs) [8,15], graphene [16,17,18], Mxene [19], and graphene oxide (GO) [20]. Moreover, when they are stuck in a single mode, traditional wearables miss the richness of the body’s language. They can feel a squeeze or sense a chill, but the whispers of heart rate, moisture, and chemical composition fall on deaf ears. E-skins, with their multimodal talents, promise to decode the full story of our biology [21].

Among gel-based conductors, hydrogels, which have remarkable tensile and conductive properties have established themselves as leading sensing materials for flexible wearable electronics, owing to their inherent flexibility and stretchability [3]. While inherently flexible and conductive, hydrogels have one vulnerability: their dependence on water, as hydrogel-based sensors lose their sensing properties at temperatures below 0 °C or above 100 °C [22]. To overcome the thermal limitations of hydrogels, the researchers replaced water with organic solvents to prepare organogels. These intrepid materials stand unfazed by both icy chills and scorching heat, expanding their realm of potential applications [23,24]. Organic solvents, despite their thermal advantages, cast a shadow of toxicity over organogels. Their long-term contact with the human body raises concerns, hindering their potential in biomedical and wearable sensor applications [25].

Unlike volatile organic solvents, ionic liquids (ILs) possess negligible vapor pressure, rendering them environmentally friendly and safer for handling. Moreover, their lack of volatility minimizes evaporation and ensures stable performance [26,27]. Ionogels are composite materials formed by infusing ILs as solvents into a pre-existing three-dimensional polymer network. This process retains the structural integrity and flexibility of the polymer while incorporating the advantageous properties of ILs, such as high conductivity, low vapor pressure, and a wide electrochemical window [28]. These excellent properties led to the widespread use of ionogels in electroluminescent devices [29,30,31], supercapacitors [32,33], transistors [34,35], and so on. The traditional conductive materials used in e-skins often fall short when it comes to mimicking the supple and dynamic nature of human skin. Ionogels, with their ionic conduction and inherent flexibility, bridge this gap, offering the potential for e-skins that move and sense with a natural fluidity, seamlessly integrating with the human body [36]. Hu et al. prepared an ionogel by immersing the polymer elastomer, poly(2-[[(butylamino)carbonyl]oxy]ethyl acrylate) (PBACOEA), in the 1-Ethyl-3-methylimidazolium bis(trifluoromethylsulfonyl)imide ([C_2_mim][NTf_2_]) [37]. The high-performance ionogel could function as a wearable sensor that can detect various movements and temperatures of the human body. The impregnation method is the simplest way to prepare ionogels, allowing the direct use of cross-linked polymer matrices. However, due to the impossibility of precisely controlling the amount of absorbed IL, the ionogels may face the risk of IL leakage [38]. In addition, the ionogels have poor tensile and fatigue resistance properties, and there is an obvious hysteresis after long-term loading [39,40].

**Scheme 1:**
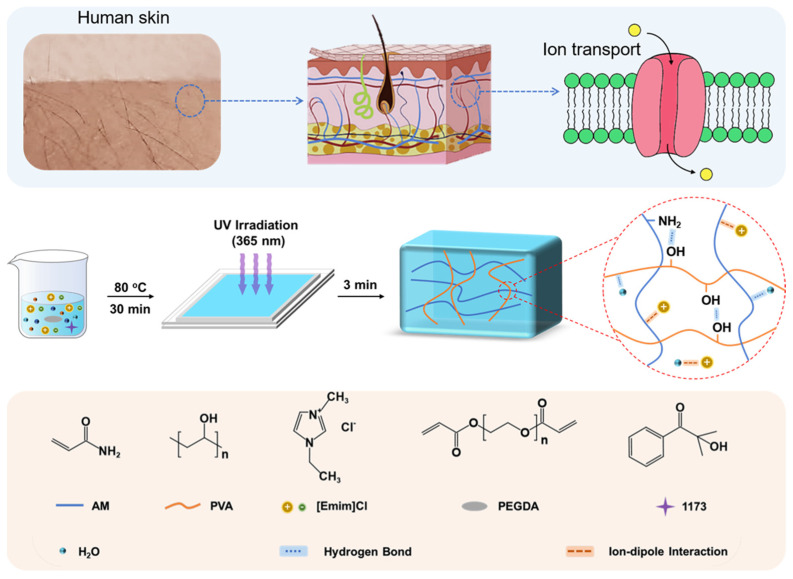
The preparation process of the PAM/PVA DN ionogels.

In this work, inspired by the multimodal sensing ability generated by ion transport in human skin, we prepared polyacrylamide/polyvinyl alcohol (PAM/PVA) DN ionogels with adjustable mechanical properties and conductivity using one-step photopolymerization (Scheme 1). This ionogel had a unique DN structure. PVA formed the first network by physical cross-linking through hydrogen bonds that act as sacrificial connections, sacrificing themselves under stress to dissipate energy and protect the overall structure. Acrylamide (AM), on the other hand, formed the second network via irreversible chemical cross-links through photopolymerization in the presence of a photoinitiator. This robust network acted as the backbone, providing strength and stability. The inherent compatibility between the IL and polymer matrix not only endowed these ionogels with exceptional transparency but also ensured their structural integrity, effectively preventing any leakage of IL. Moreover, these ionogels exhibited a wide temperature range for practical applications, minimal hysteresis effects, and remarkable repetitive adhesion strength. From accurately tracking large-scale joint movements to capturing subtle physiological signals such as pulse rate and facial expressions like frowns, these high-performance ionogels demonstrated outstanding capabilities as strain sensors. They even extended their functionality to capturing the intricate nuances of sign language.

## 2. Materials and Methods

### 2.1. Materials

The AM (99%), photoinitiator 2-hydroxy-2-methylpropiophenone (1173, 99%), and cross-linker poly(ethylene glycol) diacrylate (PEGDA, average Mw = 200 g mol^−1^) were purchased from J&K Scientific Co., Ltd (Beijing, China). The IL [Emim]Cl (98%) was purchased from Shanghai Aladdin Biochemical Technology Co., Ltd (Shanghai, China). The PVA (average degree of polymerization = 1750 ± 50) was purchased from Tokyo Chemical Industry Co., Ltd (Tokyo, Japan). All chemicals were used directly without further purification.

### 2.2. Preparation of PAM/PVA DN Ionogels and Hydrogels

Ionogels: The ionogels are referred to as PAM_x_/PVA_y_-z, where x is defined as the mass fraction of AM to AM and IL (wt%), y is defined as the mass ratio of PVA to AM (%), and z is defined as the mass ratio of PEGDA to AM (%). As an example of PAM_40_/PVA_6_-1, the preparation process of ionogels was as follows. Firstly, AM (1.6 g), [Emim]Cl (2.4 g), 1173 (0.016 g), PEGDA (0.016 g), and H_2_O (0.4 g, 1/4 of the AM mass) were mixed and stirred at 80 °C for 20 min. Then, PVA (0.096 g) was added to the above solution, and the stirring was continued for 10 min to form a homogeneous and transparent precursor solution. The precursor solution was transferred into a mold, which was placed between two pieces of glass, and after it was cooled down to room temperature, polymerization was initiated under 365 nm UV light for 3 min to obtain the PAM/PVA DN ionogel.

Hydrogels: Firstly, AM (1.6 g), 1173 (0.016 g), PEGDA (0.016 g), and H_2_O (2.4 g) were mixed and stirred at 80 °C for 20 min. Then, PVA (0.096 g) was added to the above solution, and the stirring was continued for 10 min to form a homogeneous and transparent precursor solution. The precursor solution was transferred into a mold, which was placed between two pieces of glass, and after it was cooled down to room temperature, the polymerization was initiated under 365 nm UV light for 2 h to obtain the PAM/PVA DN hydrogel.

### 2.3. Characterization

An electromechanical universal testing machine (E44.104, MTS SYSTEMS, Beijing, China) was used to characterize the mechanical and adhesion properties of the ionogels. Electrochemical impedance spectroscopy was obtained on an electrochemical workstation (CHI 760E, CH Instruments, Shanghai, China) to test the electrical resistance of the ionogels. The ionogels had a radius of 6 mm and a thickness of 2 mm. The sample was held between two stainless-steel electrodes, and to prevent deformation of the sample from affecting the results, the sample was secured between the electrodes with a hollow silicone gel pad. The AC amplitude was set to 5 mV and the test frequency was set to 1–105 Hz, and then five measurements were taken for each set of samples. The conductivity of the ionogels was then obtained using Formula (1):

(1) $$\sigma =L/R_{b}S$$

where *L* is the thickness of the ionogels; *R_b_* is the resistance of the ionogels, which is the intersection of the curve with the x-axis in the electrochemical impedance spectrum; and *S* is the area of the ionogels. Copper wires were attached to both sides of the ionogel, clamped to the ends of the electromechanical universal testing machine, and connected to an electrochemical workstation, which could be assembled into a simple system that could be used to test the electro-mechanical properties of the ionogel. The transmittance spectrogram of the ionogels was characterized using a UV-Vis spectrophotometer (Shimadzu UV-3600, SHIMADZU, Tokyo, Japan). The microscopic morphology of the ionogels was characterized using scanning electron microscopy (SEM) (TESCAN MIRA LMS, TESCAN, Brno, Czech Republic). Fourier transform infrared (FT-IR) spectra of AM, PVA, [Emim]Cl, and ionogels were obtained using an infrared spectrometer (Nicolet iS10, Thermo Fisher Scientific, Shanghai, China) in the ATR mode of testing with a wavenumber range of 400–4000 cm^−1^. The chemical migration of the electron binding energy of the surface elements of the ionogels was characterized using X-ray photoelectron spectroscopy (XPS) (Escalab250Xi, Thermo Fisher Scientific, Shanghai, China). Thermal analyses of the ionogels were obtained using a thermogravimetric analyzer (TGA) (TG/DTA8122, Rigaku, Tokyo, Japan) and a differential scanning calorimeter (DSC) (DSC 200 F3, Netzsch, Bavaria, Germany). The rheological properties of the ionogels were characterized using a rheometer.

## 3. Results and Discussion

### 3.1. Characterization of the DN Ionogels

**Figure 1:**
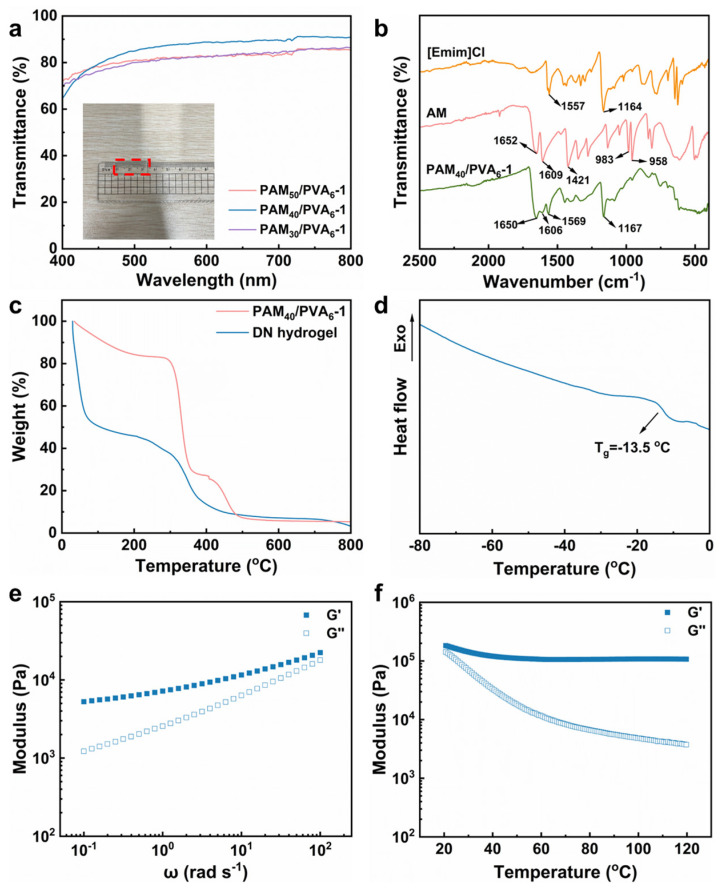
(**a**) Transmittance spectrum of ionogels with different IL content in the visible wavelength range. Inset: Photograph of PAM40/PVA6−1 placed on a ruler. (**b**) FT-IR spectra of the [Emim]Cl, AM, and PAM40/PVA6−1. (**c**) TGA curves of the PAM/PVA DN ionogel and hydrogel. (**d**) DSC curve of the PAM40/PVA6−1. (**e**,**f**) Rheological properties of PAM40/PVA6−1 with the variation in G′ and G″ with angular frequency and temperature.

Flexible DN ionogels were prepared using a simple and fast one-step photopolymerization method. AM was chosen as the polymerization monomer due to the high stretchability of polyacrylamide (PAM). When heated to 80 °C, the AM, the cross-linker PEGDA, the photoinitiator 1173, the IL [Emim]Cl, and the PVA formed a homogeneous and transparent precursor solution. The precursor solution was then poured into a mold placed between two pieces of glass. After UV irradiation of 365 nm for 3 min, a flexible DN ionogel was formed. Superior stability is crucial for practical applications. As shown in Figure S1, no IL leakage was observed when the ionogels were sheared, pressed, and stretched, indicating that the ionogels had excellent stability without the risk of IL leakage. This remarkable stability can be attributed to the compatibility between [Emim]Cl, AM, and PVA as well as the homogeneous dispersion of [Emim]Cl within the polymer network, which is evident from the SEM image (Figure S2). Furthermore, the ionogels exhibited excellent optical transparency owing to the favorable compatibility between the IL and polymer networks. The results depicted in Figure 1a demonstrated that as the content of IL increased from 50 wt% to 70 wt%, the average transmittance of the ionogels (2 mm thickness) in the visible wavelength range (400–800 nm) was 82.0%, 86.3% and 81.6%, respectively, which indicated that the ionogels had high optical transparency, which could provide a good basis for various applications in the optical field (such as smart electronic screens, electroluminescent displays and human–machine interfaces) [41,42].

The microstructure of the ionogel was characterized using FT-IR spectra to demonstrate the successful synthesis of the ionogel. The FT-IR spectra of [Emim]Cl, AM, and PAM_40_/PVA_6_−1 are presented in Figure 1b. In [EMIM]Cl, the stretching vibration peak of the C=N bond in the imidazole was observed at 1557 cm^−1^, while that of the C-N bond in the imidazole was observed at 1164 cm^−1^. These characteristic peaks were also observed in the ionogel, indicating the successful introduction of IL. Due to the presence of ion–dipole interactions, these characteristic peaks experienced a blue-shift to 1569 cm^−1^ and 1167 cm^−1^, respectively. In AM, the out-of-plane bending vibration of the terminal C-H bond in -CH=CH_2_ was observed at 958 cm^−1^ and 983 cm^−1^, respectively, and the in-plane bending vibration of the C-H bond in -CH=CH_2_ appeared at 1421 cm^−1^. However, these characteristic peaks were absent in the FT-IR spectra of the ionogel, providing evidence of the successful synthesis of PAM. Moreover, a shift from 1650 cm^−1^ to 1656 cm^−1^ was observed for the C=O bond stretching vibration. Similarly, a shift from 1608 cm^−1^ to 1613 cm^−1^ was observed for the in-plane bending vibration of the N-H bond. These shifts were accompanied by broadening of peaks, indicating enhanced intermolecular interactions and formation of hydrogen bonding between IL and polymer chains. In addition, to further investigate the chemical composition of the ionogel, the chemical shift of the electron binding energy of the surface elements of the ionogel was also characterized using XPS (Figure S3). XPS uses C1s (284.6 eV) as a reference to correct for other peaks. Four elements—C, N, O, and Cl—are observed in the total spectrum. The C1s can be divided into five subpeaks: C-C (284.6 eV), C-O (285.4 eV), C-O in PVA (286.5 eV), C-N (285.7 eV), and C=O (287.8 eV). The N1s can be divided into three subpeaks: N-H (399.4 eV), N-C (399.5 eV), and N^+^-C (401.5 eV). The O1s can be divided into two subpeaks: C=O (531.2 eV) and C-O (532.3 eV).

The same piece of PAM_40_/PVA_6_−1 was consecutively subjected to temperatures of 0, 20, 40, and 80 °C for 10 min. Remarkably, no significant changes in the size of the ionogel were observed throughout this thermal treatment, highlighting its exceptional dimensional stability across varying temperature conditions (Figure S4). To further evaluate the thermal properties of both the ionogel and hydrogel, thermogravimetric analysis (TGA) was performed. As shown in Figure 1c, the weight loss due to evaporation of water and decomposition of incompletely reacted AM dominated when the temperature was below 270 °C. Beyond this threshold, decomposition of the IL commenced, while at 390 °C, decomposition of PAM within the ionogel occurred [41]. In comparison, the PAM/PVA DN hydrogel prepared via photopolymerization exhibited a weight loss of 50% at 100 °C. Notably, the thermal stability properties demonstrated by the ionogels surpass those observed in hydrogels. The glass transition temperature (T_g_) of the ionogel was characterized using DSC. As depicted in Figure 1d, the T_g_ of the ionogel was approximately −13.5 °C, indicating its broad operational temperature range. The rheological measurements further confirmed that within the angular frequency range of 10^−1^–10^2^ rad s^−1^, the storage modulus (G′) consistently exceeded the loss modulus (G″), demonstrating solid behavior and elastic deformation of the ionogel (Figure 1e). Subsequently, the rheological investigations as a function of temperature revealed a negligible decrease in G′ from 20 to 120 °C for the ionogel. Moreover, throughout this entire temperature range, G′ remained consistently higher than G″, signifying absence of gel–sol transition for the ionogel (Figure 1f).

### 3.2. Mechanical, Conductive, and Adhesive Properties of the DN Ionogels

Mechanical properties and high electrical conductivity are essential characteristics of ionogels, enabling them to be used as flexible and stretchable conductors. Achieving exceptional mechanical properties necessitates a significant degree of cross-linking; however, increased cross-linking often leads to reduced conductivity. Consequently, researchers have directed their efforts towards enhancing the mechanical properties while preserving the conductivity of ionogels. The ionogels with adjustable mechanical properties and conductivity were synthesized by adjusting the mass ratio of IL to AM, PVA content and PEGDA content, and the effects of these factors on the mechanical properties and conductivity of the ionogels were investigated.

**Figure 2:**
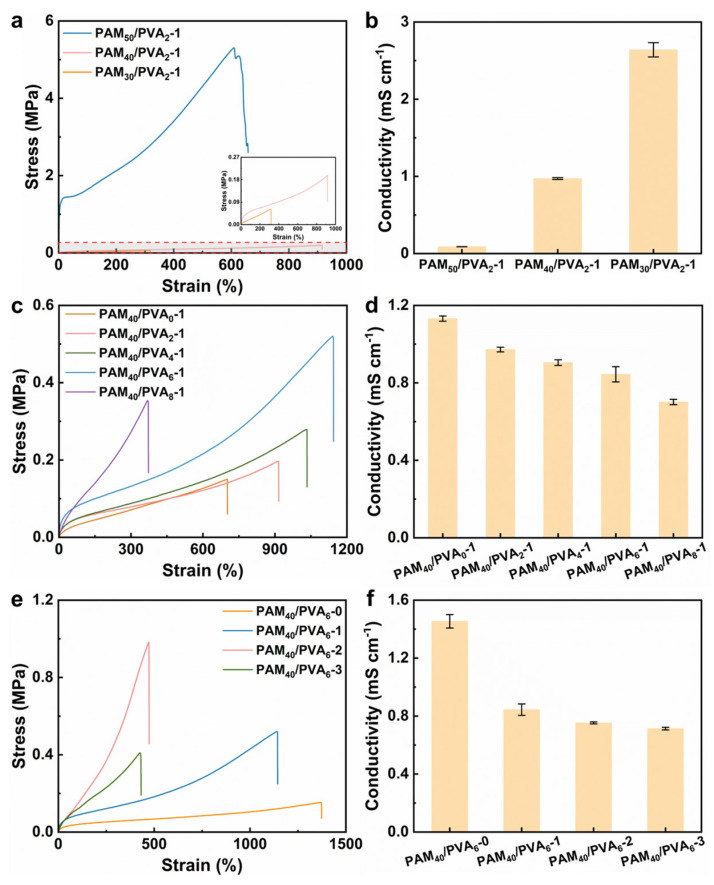
The stress−strain curves and ionic conductivity of the ionogels as a function of (**a**,**b**) IL content, (**c**,**d**) PVA content, and (**e**,**f**) PEGDA content.

Firstly, the influence of IL content on the properties of ionogels was investigated by varying the IL content to 50 wt%, 60 wt%, and 70 wt%. As the IL content increased, there was a corresponding decrease in AM content, resulting in a reduction in both the PAM cross-linked network and the mechanical properties of the ionogels (Figure 2a and Figure S5a). However, an increase in IL content led to enhanced mobility of conductive ions within the ionogels, consequently leading to an increase in conductivity (Figure S5b and Figure 2b). Secondly, the influence of PVA content on the properties of ionogels was investigated. The IL content was maintained at 60 wt%, while the PVA content was systematically adjusted to 0%, 2%, 4%, 6%, and 8% relative to the mass of AM. The Young’s modulus of the ionogels increased from 0.13 MPa to 1.02 MPa, the tensile strength from 0.15 MPa to 0.52 MPa, and the fracture elongation from 702% to 1144% as the PVA content increased from 0% to 6%. The augmentation of PVA content led to increased formation of a first cross-linking network, further enhancing the mechanical properties of the ionogels. However, when the PVA content reached 8%, a significant decrease in mechanical properties was observed due to agglomeration of molecular chains caused by a large number of hydroxyl groups present in PVA, resulting in non-uniform dispersion within the system (Figure 2c and Figure S6a). The conductivity of the ionogels gradually decreased with the increase in PVA content, ranging from 1.13 mS cm^−1^ to 0.70 mS cm^−1^, as depicted in Figure S6b and Figure 2d. This phenomenon can be attributed to the enhanced cross-linking degree of the ionogels at higher PVA contents, which hinders the mobility of ions within the system. Finally, the effect of PEGDA content on the properties of the ionogels was investigated by keeping the IL content at 60 wt% and the PVA content at 6% while varying the PEGDA content to 0%, 1%, 2%, and 3% of the AM mass, respectively. The cross-linking degree of the ionogels increased with the increase in PEGDA content, resulting in enhanced tensile strength but reduced elongation at break (Figure 2e and Figure S7a). Moreover, higher cross-linking degrees impeded ion movement, leading to a decrease in conductivity (Figure S7b and Figure 2f). The optimal formulation of the ionogel, considering its mechanical properties and conductivity, was determined to be 60 wt% for IL, 6% of AM mass for PVA, and 1% of AM mass for PEGDA (PAM_40_/PVA_6_−1). This formulation was subsequently employed for performance tests.

**Figure 3:**
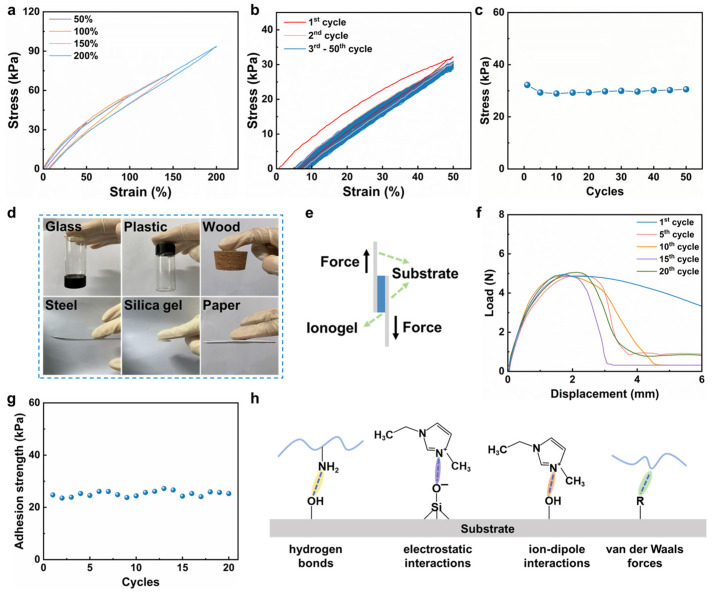
(**a**) Cyclic tensile testing of ionogels at different strains. (**b**) Cyclic tensile testing of ionogels at a strain of 50% for 50 cycles. (**c**) Maximum tensile stress during cyclic tensile testing. (**d**) Photographs of ionogels adhered to different substrates. (**e**) Schematic diagram of lap–shear test for ionogels. (**f**) Typical lap–shear curve of the ionogels. (**g**) The repeated adhesion strength of the ionogels. (**h**) The possible interactions between ionogels and substrates.

In addition to possessing excellent mechanical properties and high conductivity, the utilization of ionogels also requires them to exhibit fatigue resistance and low hysteresis. Cyclic tensile tests were conducted on the ionogels, revealing their ability to rapidly recover at various strains (Figure 3a). The dissipated energy of the ionogel gradually increased with the increase in strain (Figure S8a). To further demonstrate the stability of the ionogel under cyclic stretching, the ionogel was subjected to 50 cycles of cyclic stretching at a strain of 50%, as shown in Figure 3b. Figure S8b reveals that after the initial stretching cycle, there was a significant decrease in the amount of dissipated energy, indicating hydrogen bond breakage within the ionogel structure. However, as more stretching cycles were performed, no substantial change in dissipated energy was observed. These findings demonstrate that PAM/PVA DN ionogels possess remarkable toughness and fatigue resistance characteristics. Notably, even after undergoing 50 cyclic tensile processes, the ionogel still exhibited low hysteresis with a residual strain of approximately 9%. Furthermore, there was no significant reduction in strength during the stretching process; the maximum tensile stress only decreased by 4.97% from 32.2 kPa to 30.6 kPa (Figure 3c). The ionogel exhibits excellent mechanical properties and fatigue resistance due to the presence of covalent bonds, non-covalent bonds, and ion–dipole interactions. In this system, the physically crosslinked PVA network acts as a sacrificial network, providing an energy dissipation mechanism by breaking hydrogen bonds during stretching. On the other hand, the chemically crosslinked PAM network serves as a reinforcing network, enhancing the tensile properties of the ionogels. Additionally, incorporating DN into the structure not only improves toughness and fatigue resistance but also reduces hysteresis. These anti-fatigue properties contribute to long-term stability and minimize fracture risk in practical applications. Moreover, low hysteresis enables rapid recovery of the polymer network, resulting in a fast response performance that reduces delays in electrical signal transmission and facilitates efficient human–computer interaction.

The favorable adhesion properties of ionogels improve the stability of wearable sensors during use and make the sensing signals more reliable. As can be seen in Figure 3d, the ionogels exhibited strong adherence to diverse substrates, including glass, plastic, wood, steel, silica gel, paper, and rubber gloves. The adhesion strength of ionogels on glass substrates was evaluated using a lap–shear test method with a size of 1 × 2 cm^2^. The testing schematic is illustrated in Figure 3e. Remarkably, even after 20 repeated tests (Figure 3f,g), no significant decrease was observed in the adhesion strength of ionogel, which maintained an average value around 25.2 kPa. These results demonstrate outstanding repeatability performance for adhesive properties, which can be attributed to the abundant presence of functional groups on the surface of ionogels that facilitate various interaction forces with substrates such as hydrogen bonds, electrostatic interactions, ion–dipole interactions, and van der Waals forces, as shown in Figure 3h.

### 3.3. Sensing Performances of Ionogels

When a piece of ionogel was incorporated into a closed circuit as a conductor, it successfully illuminated a light-emitting diode (LED), thereby confirming its excellent conductivity. Upon heating the ionogel to 80 °C, the LED exhibited enhanced brightness, indicating that the conductivity increased with the temperature. Subsequently, cooling the ionogel back to 25 °C restored the LED brightness to its original level. Furthermore, subjecting the ionogel to mechanical strain resulted in reduced LED brightness, suggesting diminished conductivity; however, unloading the applied strain promptly restored the initial LED brightness (Figure S9). These intriguing observations highlight the remarkable temperature–conductivity and strain–conductivity response capabilities of the ionogels.

**Figure 4:**
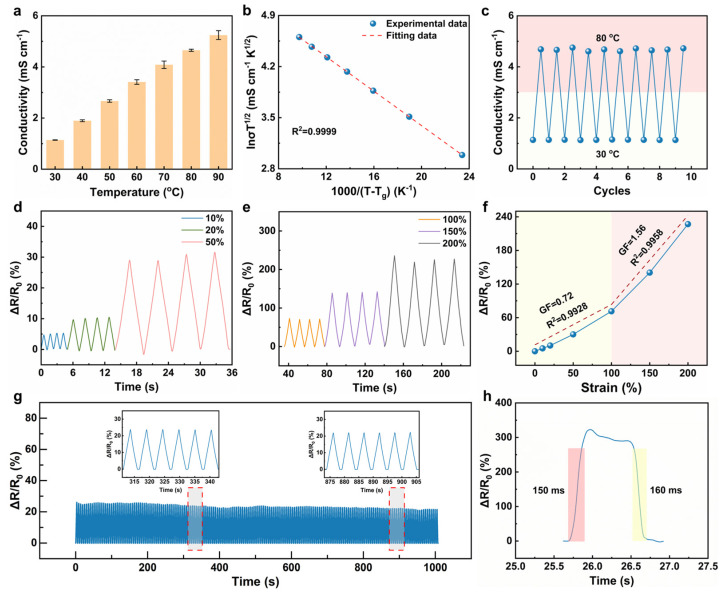
(**a**) Conductivity of ionogels at different temperatures. (**b**) VTF plot of conductivity of ionogels. (**c**) The conductivity of ionogels under alternating temperature changes at 30 °C and 80 °C. (**d**,**e**) Resistance changes of ionogels with time at different strains during the loading and unloading process. (**f**) Resistance changes of ionogels at different tensile strains. (**g**) Resistance change as a function of time for ionogels with 35% strain within 1000 s. (**h**) The response time and recovery time of ionogels.

The conductivity of the ionogel was measured at different temperatures and the results are shown in Figure 4a. As the temperature increased, there was an increase in the rate of movement of free ions within the ionogel, leading to a gradual increase in the conductivity. Notably, the observed conductivity behavior adheres to the Vogel–Tammann–Fulcher (VTF) equation:

(2) $$\sigma =AT^{-0.5}\exp[-B/(T-T_{0})]$$

where *A* is the pre-exponential factor, *B* is the activation energy, *T* is the temperature, and *T*_0_ is the ideal *T_g_* (Figure 4b) [42]. More significantly, the conductivity of the ionogel remained consistently stable even when subjected to alternating temperatures between 30 °C and 80 °C, as shown in Figure 4c, thereby demonstrating its wide-ranging applicability as well as its exceptional cycling stability across different temperature ranges. These findings strongly suggest that the ionogel holds promising potential for utilization in temperature sensing applications.

The electromechanical properties of the ionogels were evaluated using the relative change in resistance (Δ*R*/*R*_0_) at various strains, where Δ*R* is the change in resistance and *R*_0_ is the initial resistance. As can be seen from Figure 4d,e, during stretching or unloading, the resistance of the ionogels exhibited a gradual increase or decrease due to continuous variations in the cross-sectional area, demonstrating remarkable periodicity. Additionally, the ionogel exhibited an outstanding linear relationship between the relative change in resistance and strain, which is advantageous for practical applications as it eliminates the need for complex correction processes and enhances sensing signal accuracy and sensitivity. The sensitivity of strain sensors can be characterized by the gauge factor (GF), where a higher GF indicates greater sensor sensitivity. The GF can be computed according to the following equation:

(3) $$GF=\left(\Delta R/R_{0}\right)/(\Delta L/L_{0})$$

where Δ*L* is the change in length and *L*_0_ is the initial length. It is evident that the GF reached 0.72 within a strain range of 0–100% and increased to 1.56 within a strain range of 100–200%, which proves that the ionogel has a high sensitivity, as shown in Figure 4f. Then, the ionogel was cyclically stretched at a strain of 35% to evaluate its resistance change, as illustrated in Figure 4g. Remarkably, no significant decline in the sensing signal of the ionogel was observed over a duration of 1000 s, affirming its stability and durability for long-term utilization. In addition to stable sensing signals, rapid response time and recovery time are also crucial for strain sensors. As depicted in Figure 4h, the ionogel exhibited a short response time of 150 ms and a recovery time of 160 ms, respectively, indicating its exceptional responsiveness, which was attributed to the minimal hysteresis and heightened sensitivity of the ionogel. In fields such as health monitoring and machine fault detection, reduced response times enable prompt reactions and accident prevention.

The GF, response time, and recovery time of ionogels with different structures in recent years are summarized in Table 1. Compared to previous studies, the PAM/PVA DN ionogel exhibits higher sensitivity as well as faster response and recovery times.

### 3.4. The Ionogels for Monitoring Physiological Signals and Detecting Sign Language

The excellent flexibility, electrical response to strain, and sensitivity of ionogels render them highly suitable for wearable flexible strain sensors. By affixing copper wires to both sides of the ionogel, it can be employed as a simplistic wearable system that can detect human body movement signals. Due to the inherent adhesive properties of ionogel, it can adhere to human skin without detachment even in the absence of additional glue, thereby ensuring more stable sensing signals.

**Figure 5:**
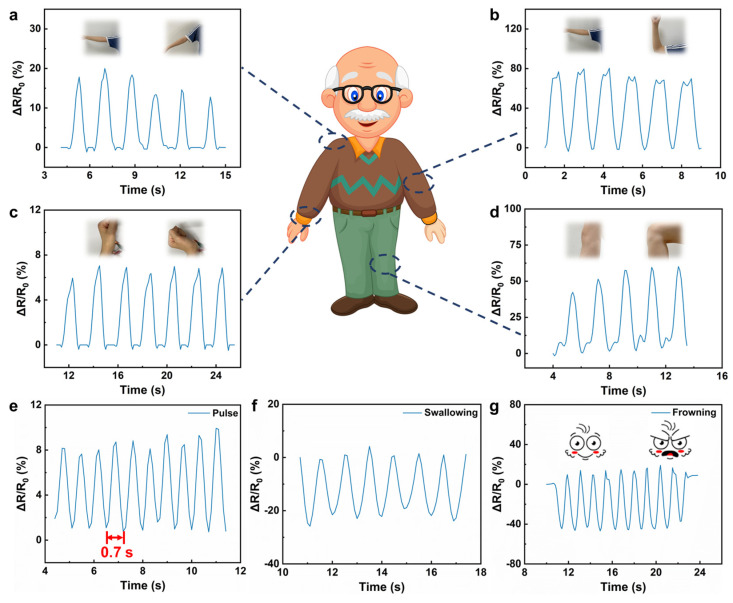
(**a**–**d**) Resistance changes of ionogels during the detection of shoulder, elbow, wrist, and knee movements. (**e**−**g**) Resistance changes of ionogels during the detection of small movements such as pulse, swallowing, and frowning.

As shown in Figure 5a, the ionogel was applied onto the shoulder, and when the shoulder was lifted at varying angles, distinct strains resulted in different rates of resistance change within the ionogel and consequently yielded diverse output electrical signals. In addition, the ionogel adhered to other articulations of the body can effectively detect joint movements, such as those occurring at the elbow, wrist, and knee (Figure 5b–d). Notably, all recorded electrical signals exhibited excellent repeatability. More importantly, owing to the high sensitivity of the ionogel, it can be utilized not only for monitoring macroscopic joint movements but also for capturing subtle motions. As illustrated in Figure 5e–g, the ionogel has demonstrated its capability in tracking physiological activities such as pulse beats, swallowing actions, and facial expressions like frowning. Figure 5e reveals a pulse beat duration of approximately 0.7 s, falling within the normal range, indicating the potential application of ionogels in health monitoring and telemedicine practices.

**Figure 6:**
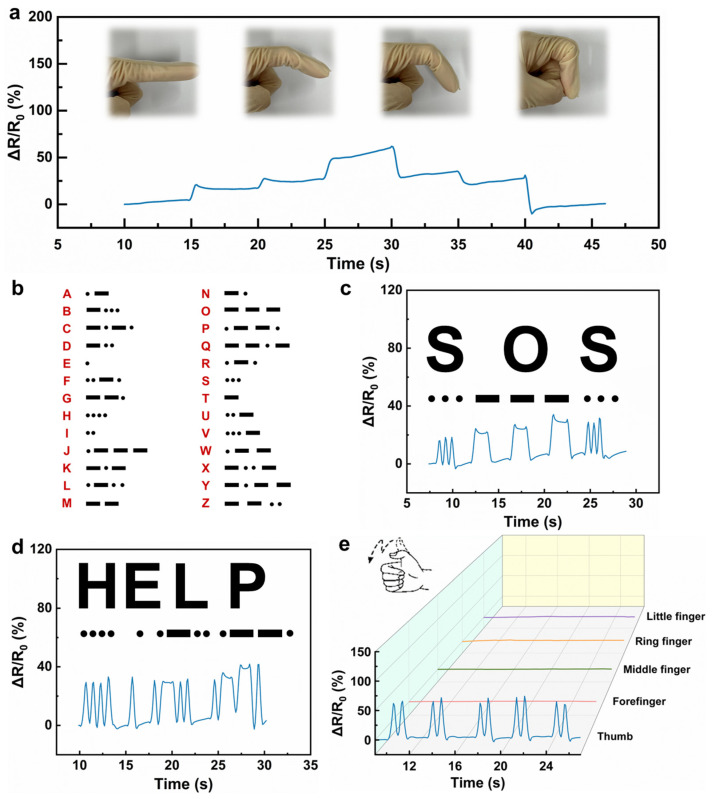
(**a**) Changes in resistance of ionogels with time during the detection of finger bending and recovery angles. (**b**) Diagram of Morse code. (**c**,**d**) Electrical signals were obtained by ionogels in the process of Morse code communication. (**e**) Changes in the resistance of ionogels adhered to each finger during sign language detection.

The finger bending and recovery angle can be detected by affixing the ionogel to the finger. As depicted in Figure 6a, when the bending angle of the finger gradually increased from 0° to 90°, there was a gradual increase in resistance due to the stretching of the ionogel. However, the resistance of the ionogel did not change significantly when the bending angle of the finger remained constant. Furthermore, during a gradual decrease in the finger bending angle, the resistance of the ionogel also gradually decreased back to its original value, thereby demonstrating that the ionogel possessed potential for detecting finger movement angles.

Morse code, widely employed as an early wireless communication medium, comprises a distinct arrangement of dots and dashes to represent various letters and numbers (Figure 6b). Based on the ability of the ionogel to detect finger movement, we used the ionogel for Morse code communication, where brief finger bending intervals corresponded to dots while prolonged finger bending intervals represented dashes. Depending on the duration of finger bending, we successfully generated “SOS” and “HELP” signals, as illustrated in Figure 6c,d.

Sign language serves as a means of communication for individuals with hearing and speech impairments. However, effective communication with non-sign language users still poses challenges. Therefore, it is particularly important to develop a sign language translator. Inspired by Morse code, in our experiment, the ionogels were employed for sign language detection. Specifically, the four fingers of the right hand were clenched into a fist while raising and subsequently bending the thumb twice (signifying “thank you”). The electrical signals of the ionogels adhered to each finger were recorded. As shown in Figure 6e, when the thumb was bent downward, an increase in resistance was observed solely within the ionogel attached to it, while no disruption or significant alteration occurred in the electrical signals originating from other fingers. This observation suggests promising prospects for utilizing ionogels in sign language detection.

## 4. Conclusions

In conclusion, DN ionogels were synthesized using [Emim]Cl, AM, and PVA, allowing for adjustable mechanical and conductive properties across a wide range. By adjusting the different ratios, the ionogels exhibited tensile strengths ranging from 0.06 to 5.30 MPa, fracture elongations ranging from 363% to 1373%, and conductivities ranging from 0.09 to 2.64 mS cm^−1^. The excellent compatibility between the IL and polymer matrix resulted in highly transparent ionogels. Furthermore, the uniform dispersion of IL within the polymer network prevented any leakage even under shear, pressure, stretching, or twisting conditions. Incorporating an additional energy dissipation mechanism enhanced fatigue resistance and reduced hysteresis in the ionogels. Additionally, these ionogels demonstrated a bi-modal response to temperature and strain with a GF of 0.72 in the range of 0–100% strain and a GF of 1.56 in the range of 100–200% strain. Finally, the ionogel was employed as a strain sensor to monitor human physiological signals and detect sign language, highlighting its vast potential in the fields of wearable electronic devices and sign language interpretation.

## Figures and Tables

**Table 1:** Comparison of ionogels with different structures in terms of GF, response time, and recovery time.

Polymer Network	ILs	GF	Response Time and Recovery Time (ms)	Ref.
WPU	[Emim][DCA]	0.53 (1–30%)	~1700/800	[43]
		0.83 (30–100%)		
		1.38 (100–200%)		
PVDF-co-HFP	[Emim][TFSI]	0.63 (20–80%)	—	[40]
		1.03 (80–300%)		
PDAAM	[Bmim][TFSI]	1.51 (0–400%)	170/190	[44]
PMMA-SiO_2_ NPs	[Bmim]PF_6_	0.39 ± 0.02 (0–50%)	3340/2880	[45]
		0.83 ± 0.2 (50–100%)		
PHFBA-r-OEGA	[Bmim][TFSI]	1 (0–200%)	300/600	[46]
Agarose/PHEA	[Emim]Cl	~1.1 (0–100%)	80	[42]
P(TFEA-co-AAm)	[Emim][TFSI]	0.83 (0–200%)	200/300	[47]
		1.38 (200–400%)		
		1.85 (400–600%)		
scPLA-PEG	[Emim]BF_4_	1.15 (0–200%)	—	[48]
		1.24 (200–300%)		
P(ACMO/UA)	[Emim][TFSI]	0.98 (0.5–100%)	65/87	[49]
		1.56 (100–300%)		
PAM/PVA	[Emim]Cl	0.72 (0–100%)	150/160	This work
		1.56 (100–200%)		

## Data Availability

The data that support the findings of this study are available from the corresponding author upon reasonable request.

## Supplementary Materials

The following supporting information can be downloaded at https://www.mdpi.com/article/10.3390/bios14050227/s1, Figure S1: Ionogels were (a) cut into pieces, (b) placed under pressure, and (c) stretched to demonstrate that there was no IL leakage; Figure S2: SEM image of the surface of PAM_40_/PVA_6_-1; Figure S3: (a) Total XPS spectrum of PAM_40_/PVA_6_-1. (b) C1s, (c) N1s, and (d) O1s spectrum of PAM_40_/PVA_6_-1; Figure S4: (a–d) Pictures of the same piece of PAM_40_/PVA_6_-1 placed successively at 0, 20, 40 and 80 °C for 10 min; Figure S5: (a) Young’s modulus, tensile strength and fracture elongation of ionogels with different IL contents. (b) Electrochemical impedance spectroscopy plots of ionogels with different IL contents; Figure S6: (a) Young’s modulus, tensile strength and fracture elongation of ionogels with different PVA contents. (b) Electrochemical impedance spectroscopy plots of ionogels with different PVA contents; Figure S7: (a) Young’s modulus, tensile strength and fracture elongation of ionogels with different PVA contents. (b) Electrochemical impedance spectroscopy plots of ionogels with different PEGDA contents; Figure S8: (a) The energy dissipated by ionogel during cyclic tensile testing at different strains. (b) The energy dissipated by the ionogel during cyclic tensile testing at 50% strain as a function of the number of cycles; Figure S9: The brightness of the LED changed with temperature and strain when the ionogel was placed in a closed circuit as a conductor.
