# Supplementary material for: Highly Stretchable Double Network Ionogels for Monitoring Physiological Signals and Detecting Sign Language
Source: Biosensors (Basel). 2024 May 3;14(5):227. doi: 10.3390/bios14050227 (PMC11118894; doi:10.3390/bios14050227)
Supplement: Supplementary file 1 [file biosensors-14-00227-s001.zip › biosensors-2962239-supplementary.pdf]

Supporting information

Highly Stretchable Double Network Ionogels for Monitoring Physiological Signals and Detecting Sign Language

Ya Jiang, Shujing Zhao, Fengyuan Wang, Xiaoyuan Zhang * and Zhiqiang Su *

State Key Laboratory of Chemical Resource Engineering, Beijing Key Laboratory of Advanced Functional Polymer Composites, Beijing University of Chemical Technology, Beijing 100029, China; 2021200427@buct.edu.cn (Y.J.); 2021400142@buct.edu.cn (S.Z.); 15832848097@163.com (F.W.)

* Correspondence: xiaoyuan.zhang@buct.edu.cn (X.Z.); suzq@mail.buct.edu.cn (Z.S.)

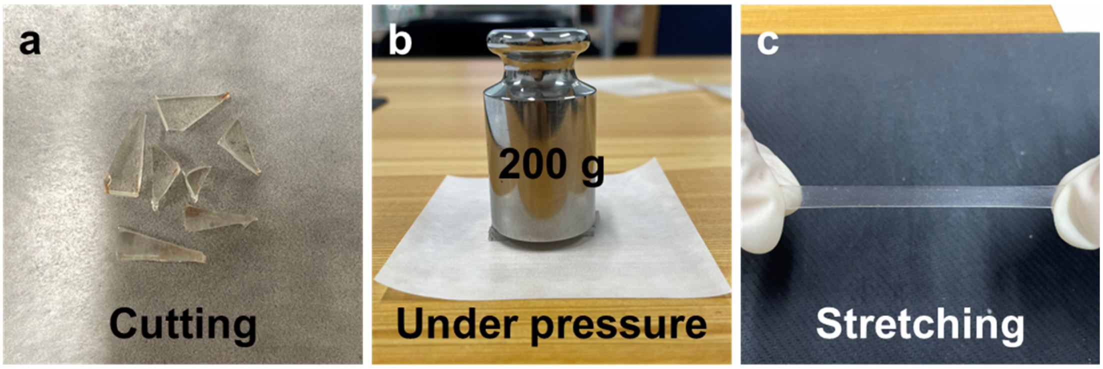

Figure S1. Ionogels were (a) cut into pieces, (b) placed under pressure, and (c) stretched to demonstrate that there was no IL leakage.

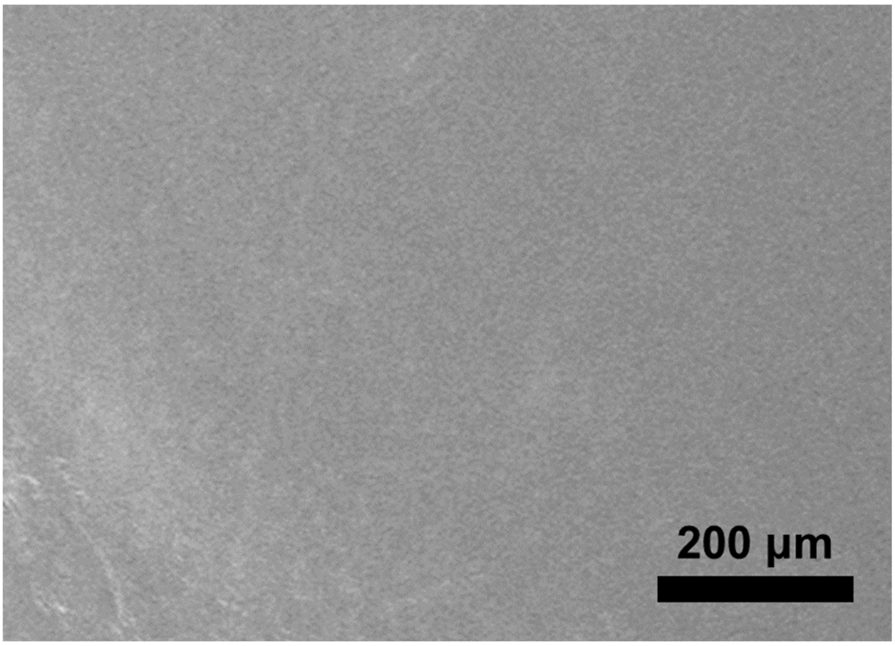

Figure S2. SEM image of the surface of PAM_{40}/PVA_6-1.

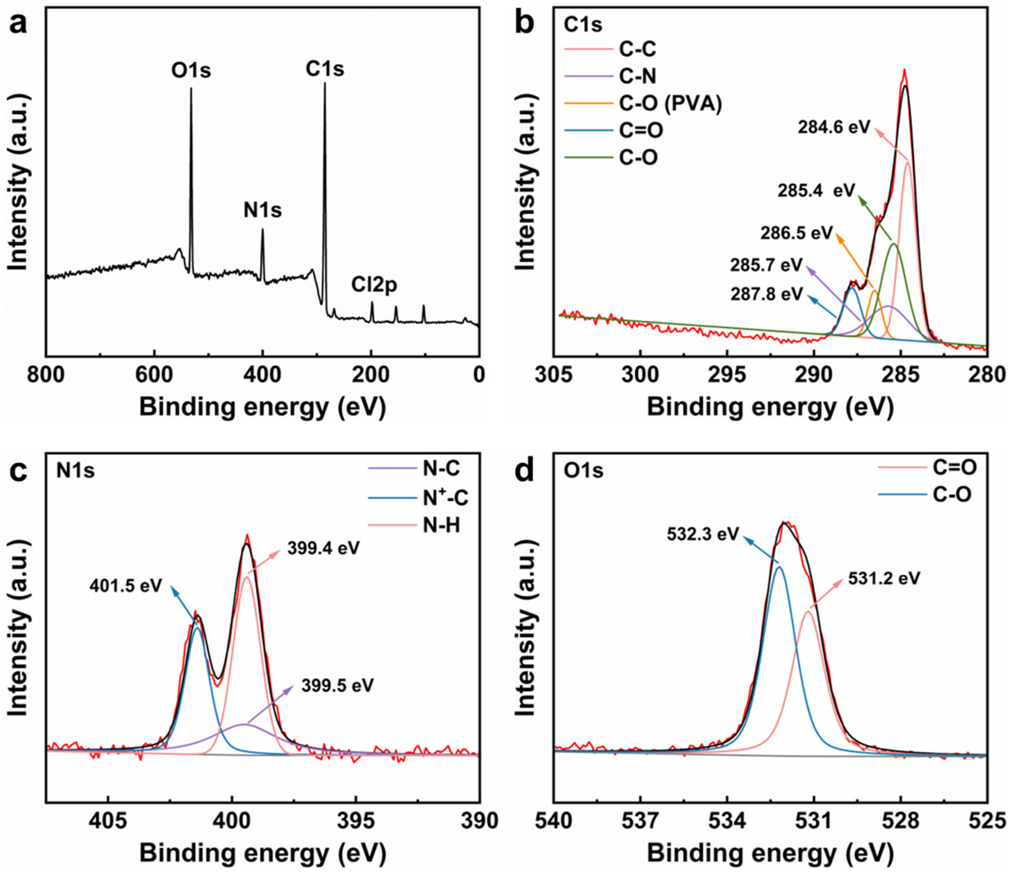

Figure S3. (a) Total XPS spectrum of PAM_{40}/PVA_{6}-1. (b) C1s, (c) N1s, and (d) O1s spectrum of PAM_{40}/PVA_{6}-1.

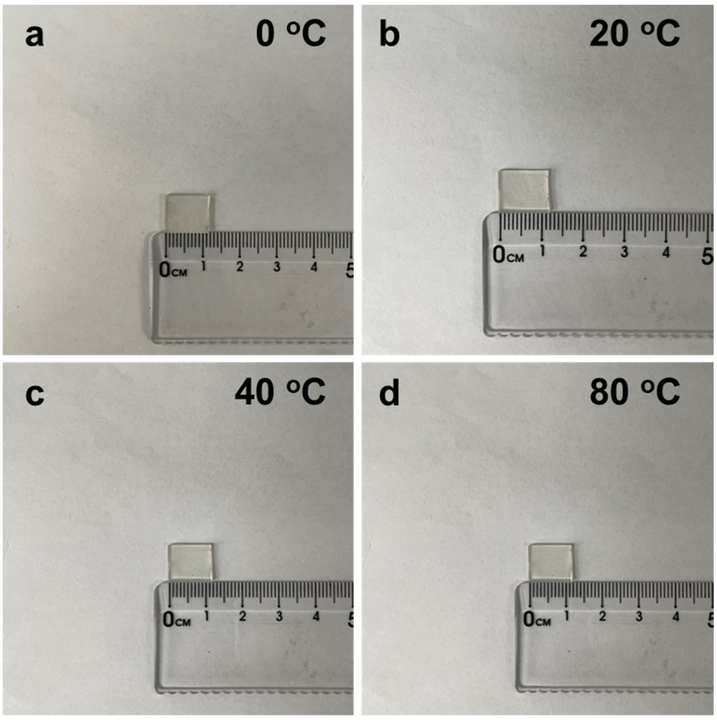

Figure S4. (a-d) Pictures of the same piece of PAM_{40}/PVA_{6}-1 placed successively at 0, 20, 40 and 80 °C for 10 min.

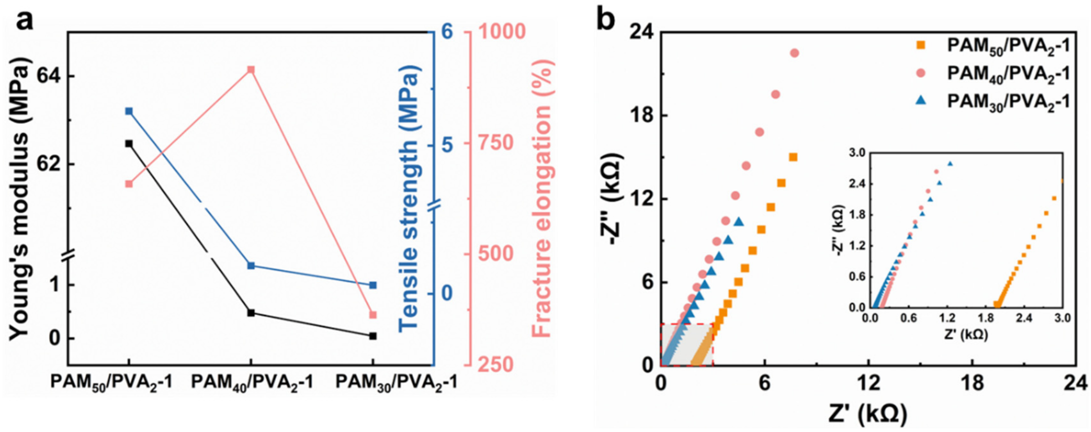

Figure S5. (a) Young's modulus, tensile strength and fracture elongation of ionogels with different IL contents. (b) Electrochemical impedance spectroscopy plots of ionogels with different IL contents.

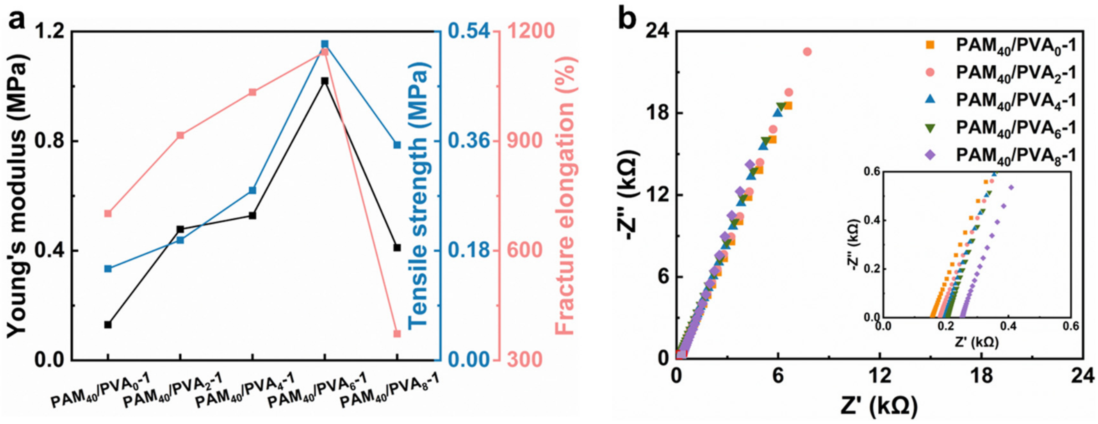

Figure S6. (a) Young's modulus, tensile strength and fracture elongation of ionogels with different PVA contents. (b) Electrochemical impedance spectroscopy plots of ionogels with different PVA contents.

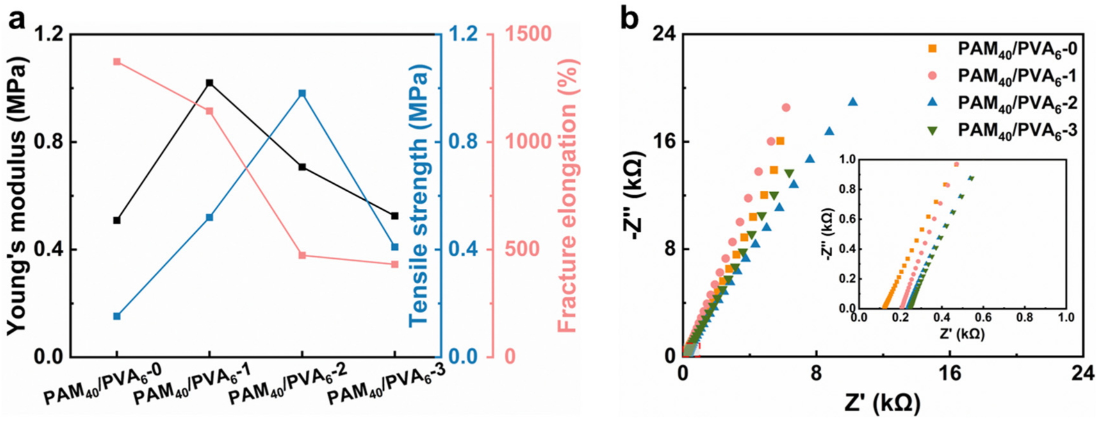

Figure S7. (a) Young's modulus, tensile strength and fracture elongation of ionogels with different PEGDA contents. (b) Electrochemical impedance spectroscopy plots of ionogels with different PEGDA contents.

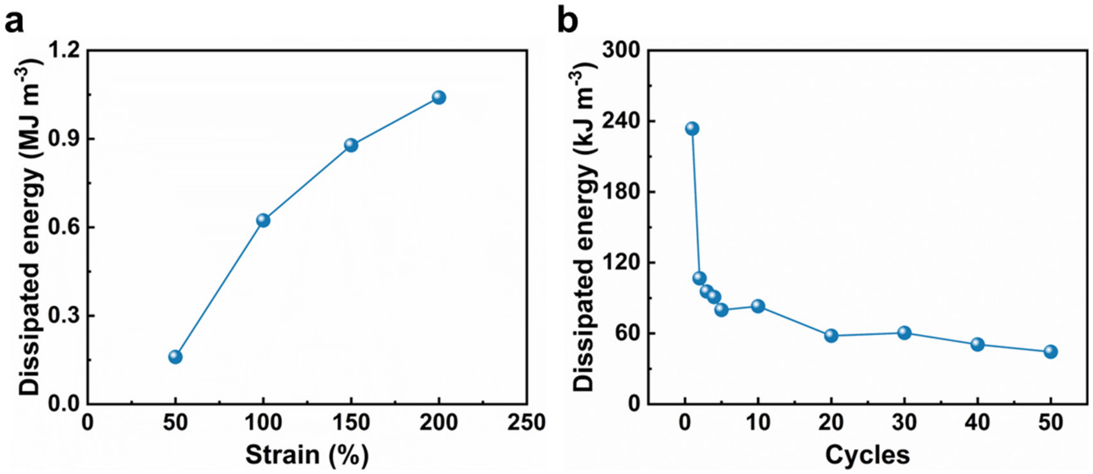

Figure S8. (a) The energy dissipated by ionogel during cyclic tensile testing at different strains. (b) The energy dissipated by the ionogel during cyclic tensile testing at 50% strain as a function of the number of cycles.

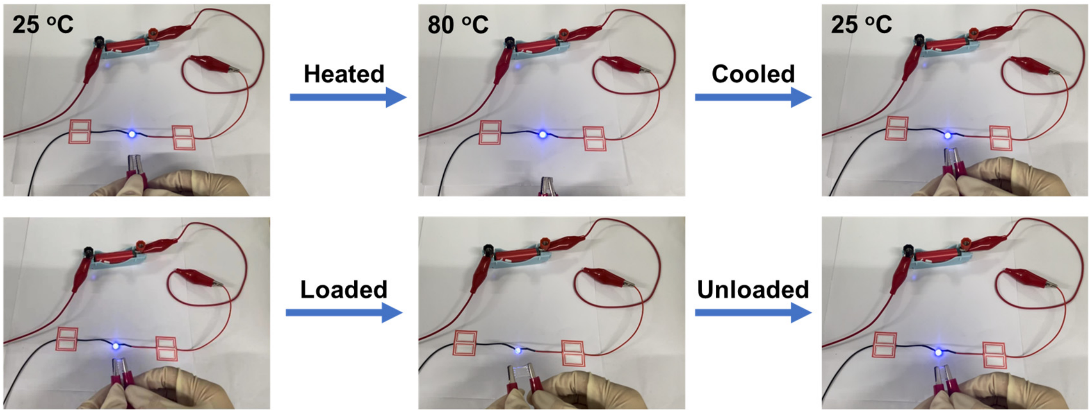

Figure S9. The brightness of the LED changed with temperature and strain when the ionogel was placed in a closed circuit as a conductor.
